# New World Pleistocene Horses: Pruning the Equid Tree

**DOI:** 10.1371/journal.pbio.0030275

**Published:** 2005-06-28

Darwin's evolutionary theories got a big boost when Othniel Charles Marsh assembled his North American horse fossils in the 1870s. They showed the gradual (presumed linear) evolution of a small, multi-toed Hyracotherium (then known as Eohippus) into the larger, hoofed modern Equus. As new fossils appeared, it became clear that the equid tree—which includes modern horses, zebras, burros, and the like—shows a complex evolutionary pattern with many branches. By the early 1900s, over 50 equid species were named for the Pleistocene (1.8 million years [Ma] to 10,000 years ago) alone.

Since then, the validity, origin, and relationships of these classifications have been revised, especially for the species of the Pliocene (5 Ma to 1.8 Ma ago) and Pleistocene epochs. In a new paper, Jaco Weinstock, Alan Cooper, and their colleagues use ancient DNA to upset conventional models describing the origins of two extinct forms: the North American “stilt-legged” horses and the stocky South American genus, Hippidion.

New World “stilt-legged” horses often appear in fossil deposits along with a second equid form that shares morphological characteristics with Eurasian caballines—a group that includes the domestic horse and the nearly extinct Przewalskii horse of Mongolia. Because stilt-legged horses had builds similar to the onager and kiang, rare wild asses that live in Asia (Asian hemionids), it's been suggested that stilt-leggeds migrated from Asia across the Bering Strait. Hippidion shows up in South American fossils about 2.5 Ma ago. It has been considered a descendant of a primitive Miocene (23.8 Ma to 5.3 Ma ago) horse that diverged from the ancestral Equus lineage about 10 Ma ago. But a recent genetic analysis of Patagonian Hippidion specimens placed the fossils within an extinct Equus lineage that migrated to South America more recently—suggesting that the specimen had been misidentified as Hippidion.

To analyze the horse fossils, Weinstock et al. used a well-established genetic technique based on mitochondrial DNA (mtDNA). Mitochondria provide most of a cell's energy needs, but it is their genome that interests evolutionary biologists—specifically, a stretch of mtDNA sequence called the control region. This region mutates at a high rate, but the patterns of mutations remain stable over thousands of generations, providing a tool for inferring evolutionary relationships.

The authors first extracted mtDNA from horse bones (mostly toes) from Eurasia and North and South America dating back to 53,000 years ago. Their genetic analysis showed that Hippidion, stilt-leggeds, and caballines all arose from a common lineage. Even though stilt-leggeds share morphological traits with the Asian hemionids, they are genetically distinct, suggesting a convergence of form rather than a common ancestry. And because none of the Old World specimens had genetic sequences similar to sequences extracted from the stilt-legged horses, it's likely that the stilt-legged horses were North American endemics. The genetic analysis also showed that stilt-legged specimens from north and south of the ice sheets that bisected North America during long stretches of the Pleistocene belong to the same taxon, suggesting a wide-ranging species. [Fig pbio-0030275-g001]

**Figure pbio-0030275-g001:**
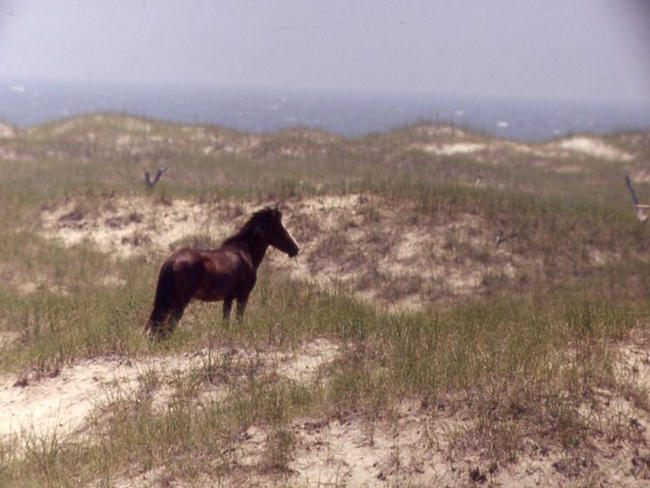
Like this wild horse from North Carolina, free-ranging American horses descended from European stock. Horses native to the New World became extinct about 10,000 years ago

Hippidion and stilt-leggeds cluster as sister taxa—in direct contrast to paleontological models of Hippidion's ancient form. The sequences are truly from Hippidion fossils (rather than the Equus lineage proposed in a recent report), the authors argue, because all the fossils came from Late Pleistocene cave deposits in southern Patagonia known to contain only Hippidion saldiasi specimens—and all but one specimen came from these deposits. Along with telling morphological and size characteristics of the bones, these results suggest that Hippidion emerged closer to 3 Ma ago.

A final analysis of horse specimens from the Pleistocene, historic, and recent caballines—which have been grouped as separate species based on their diverse size—suggests that all North American caballines may belong to the same species. Altogether, the results suggest that just two horse lineages—caballine and stilt-legged—may have lived in North America during the Late Pleistocene. Both lineages showed regional and temporal variations in size and form. Though these variations have been taken to represent many different species, the authors propose that the two lineages are more likely just two species whose variations reflect adaptations to different environments. If true, this model could provide a tool for exploring how environmental adaptations give rise to morphological variation.

